# Acetylation of PAMAM dendrimers for cellular delivery of siRNA

**DOI:** 10.1186/1472-6750-9-38

**Published:** 2009-04-23

**Authors:** Carolyn L Waite, Sarah M Sparks, Kathryn E Uhrich, Charles M Roth

**Affiliations:** 1 Department of Chemical and Biochemical Engineering, Rutgers University, Piscataway, NJ, USA; 2 Department of Chemistry and Chemical Biology, Rutgers University, Piscataway, NJ, USA; 3 Department of Biomedical Engineering, Rutgers University, Piscataway, NJ, USA

## Abstract

**Background:**

The advancement of gene silencing via RNA interference is limited by the lack of effective short interfering RNA (siRNA) delivery vectors. Rational design of polymeric carriers has been complicated by the fact that most chemical modifications affect multiple aspects of the delivery process. In this work, the extent of primary amine acetylation of generation 5 poly(amidoamine) (PAMAM) dendrimers was studied as a modification for the delivery of siRNA to U87 malignant glioma cells.

**Results:**

PAMAM dendrimers were reacted with acetic anhydride to obtain controlled extents of primary amine acetylation. Acetylated dendrimers were complexed with siRNA, and physical properties of the complexes were studied. Dendrimers with up to 60% of primary amines acetylated formed ~200 nm complexes with siRNA. Increasing amine acetylation resulted in reduced polymer cytotoxicity to U87 cells, as well as enhanced dissociation of dendrimer/siRNA complexes. Acetylation of dendrimers reduced the cellular delivery of siRNA which correlated with a reduction in the buffering capacity of dendrimers upon amine acetylation. Confocal microscopy confirmed that escape from endosomes is a major barrier to siRNA delivery in this system.

**Conclusion:**

Primary amine acetylation of PAMAM dendrimers reduced their cytotoxicity to U87 cells, and promoted the release of siRNA from dendrimer/siRNA complexes. A modest fraction (approximately 20%) of primary amines of PAMAM can be modified while maintaining the siRNA delivery efficiency of unmodified PAMAM, but higher degrees of amine neutralization reduced the gene silencing efficiency of PAMAM/siRNA delivery vectors.

## Background

Since its discovery in 1998, RNA interference (RNAi) has rapidly become a routine and powerful tool for use in basic research and has also gained momentum in development as a therapeutic [1-3]. While RNAi is an elegant, endogenous and conserved mechanism to selectively silence genes, inefficient delivery of exogenous short interfering RNA (siRNA) molecules to cells and tissues remains a barrier to its therapeutic development. As a result, the design of effective siRNA delivery systems is crucial for the clinical advancement of RNAi. Specifically, delivery vectors must be designed to effectively complex with nucleic acid molecules and aid in overcoming intracellular barriers such as endosomal escape and cytoplasmic vector dissociation.

A variety of molecules including polymers, lipids, and peptides have been studied for their effectiveness as delivery vectors for DNA and RNA molecules [4]. Successful delivery vectors must exhibit a combination of functional attributes. Polymeric carrier molecules should be cationic to complex with nucleic acids, possess a high buffering capacity, exhibit low cytotoxicity, and also contain chemically reactive groups that can be modified for the addition of targeting moieties or other groups [4,5].

Highly branched, dendritic polymers including poly(amidoamine) (PAMAM) have recently attracted interest as nucleic acid delivery vectors. Previous work has demonstrated that dendrimers can bind to DNA and RNA molecules and mediate modest cellular delivery of these nucleic acids [6-9]. Recently, some studies have evaluated the use of PAMAM dendrimers for successful delivery of siRNA or antisense molecules. Generation 5 dendrimers were found to have poor cellular delivery of siRNA to NIH 3T3 MDR cells, though they mediated moderately effective delivery of antisense oligonucleotides [10]. Another study found that increasing the PAMAM dendrimer generation to seven to increase the number of primary amine groups significantly enhanced siRNA delivery efficiency, possibly by enhanced amine-induced pH buffering[11]. However, cytotoxicity of highly cationic dendrimers is a marked problem that hinders their widespread use in drug and gene delivery [6,7].

Thus, it is desirable to exploit the potential of PAMAM dendrimers for nucleic acid delivery applications while reducing their cytotoxicity. The cytotoxicity of dendrimers can be reduced by conjugating hydrophilic polymers to the periphery of the dendrimer [12-14], by conversion of a fraction of the cationic amine groups to uncharged moieties [15-17], or by modifying a neutrally charged dendrimer with a few cationic amino acid groups sufficient to facilitate nucleic acid complexation[18]. Since a wide variety of modifications exist to alter the properties of dendrimers, the development of structure-activity relationships will accelerate determination of the optimal dendrimer properties for a particular application [19-21].

In this study, the effect of primary amine acetylation of the cationic, dendrimeric polymer, PAMAM, on siRNA delivery was analyzed. Previous studies have shown that neutralizing charges on the cationic polymer, polyethylenimine (PEI), by amine acetylation enhanced the transfection efficiency of plasmid DNA [22,23]. This marked improvement in transfection efficiency correlated with decreased polymer/DNA interactions, thus promoting intracellular unpackaging of DNA from the polymer [23]. Furthermore, amine acetylation of cationic polymers is attractive since it has been shown to reduce cytotoxicity in a variety of different cell lines [16,23]. We studied dendrimer/siRNA interactions as well as the ability of acetylated dendrimers to deliver siRNA to cells and elicit a gene silencing effect. In addition, we evaluated the tradeoff between reduced polymer/siRNA interactions and reduced endosomal buffering capacity. These design parameters are important in the rational modification of PAMAM dendrimers for siRNA delivery.

## Results

### Partial acetylation of PAMAM dendrimers

**Figure 1:**
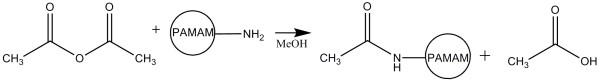
**Acetylation of PAMAM dendrimers**. Acetic anhydride reacts with primary amines of G5 PAMAM dendrimers to produce acetylated PAMAM.

The primary amines of generation 5 (G5) PAMAM dendrimers were acetylated by reaction with prescribed amounts of acetic anhydride as depicted in Figure 1. ^1^H NMR analysis of acetylated dendrimers was performed using a method similar to that described previously[24]. Briefly, the fraction of primary amine acetylation was determined by comparing the intensity of the peak at 1.87 ppm corresponding to -CH_3 _protons of the acetyl group to the sum of all -CH_2_-peaks. The observed extents of amine acetylation were very close to the theoretical maxima indicated by reaction stoichiometry (Table 1). The acetylated dendrimers are denoted by their experimentally determined acetylation levels as Ac_20_, Ac_40_, Ac_60_, and Ac_84_.

**Table 1:** Extent of primary amine acetylation as determined by ^1^H NMR spectroscopy.

**Sample**	**(-CH_3_/-CH_2_-) ratio**	**Number of acetyl groups added**	**Percent of primary amine acetylation**
Ac_20_	0.041	27	21.1
Ac_40_	0.075	50	39.1
Ac_60_	0.115	76	59.4
Ac_84_	0.16	107	83.6

### PicoGreen dye exclusion

**Figure 2:**
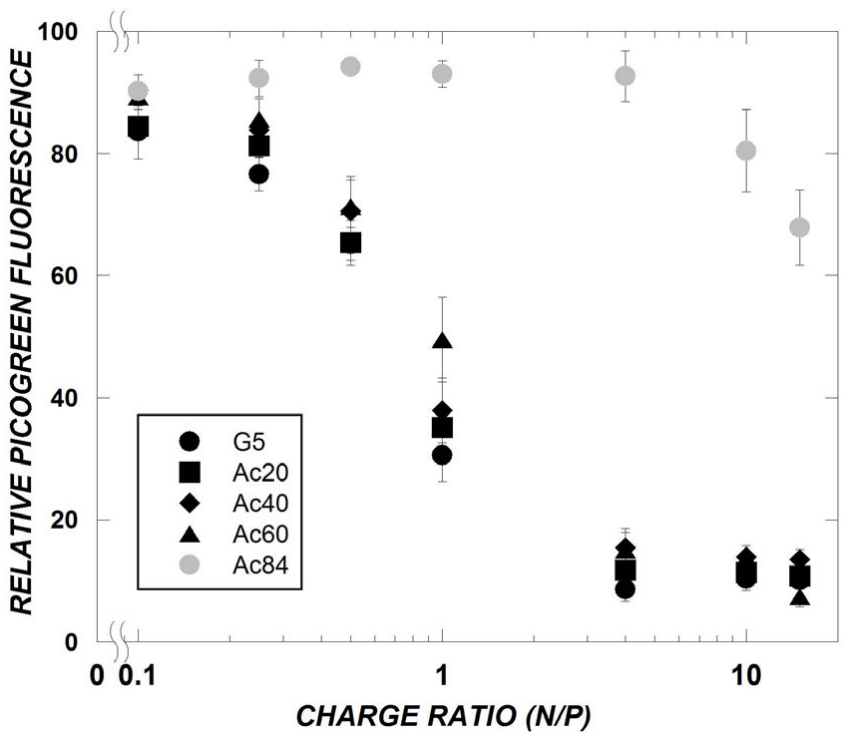
**Effect of amine acetylation on siRNA complexation by PicoGreen dye exclusion**. Complexes were prepared in PBS at a final siRNA concentration of 2 μg/mL. PicoGreen fluorescence correlates to unbound siRNA present in solution. Data represent mean ± SEM (*n *= 3).

The ability of acetylated dendrimers to complex with siRNA as a function of polymer/siRNA charge ratio was evaluated. The amount of unbound siRNA in solutions of dendrimer/siRNA was determined by measuring the fluorescence of a commercially available dye, PicoGreen, that fluoresces upon binding to double-stranded DNA or RNA. The fluorescence intensity decreased when increasing amounts of polymer were added to a fixed amount of siRNA, indicating association of siRNA with the polymer (Figure 2). At N/P = 1, differences in siRNA complexation ability could be discerned, with higher fractions of siRNA forming complexes with dendrimers possessing the greater primary amine levels. However, by N/P = 10, almost complete complexation of siRNA was observed for unmodified dendrimer (G5), Ac_20_, Ac_40_, and Ac_60_. In contrast, the binding curve for the Ac_84 _polymer was shifted markedly to the right, and little siRNA complexation was observed at N/P = 10. Since the Ac_84 _dendrimer was not able to complex with siRNA at N/P = 10, it was excluded from further studies.

### Complex size and zeta potential

Having demonstrated that acetylated dendrimers are able to complex with siRNA, the characteristics of these complexes were evaluated further by DLS and zeta potential measurements. Particle size analysis by DLS showed the formation of ~200 nm complexes between dendrimers and siRNA, regardless of acetylation extent (Table 2). Somewhat surprisingly, zeta potential measurements were approximately equal (~40 mV) for siRNA complexes with G_5_, Ac_20_, and Ac_40 _dendrimers. Though we do expect the surface charge of these complexes to become less cationic upon primary amine acetylation, this trend may not be reflected in zeta potential measurements, as surface charge has been shown to be non-linearly correlated to zeta potential [25]. However, while the zeta potential of dendrimer/siRNA complexes at N/P = 15 for G5, Ac_20_, and Ac_40 _were approximately equal (~40 mV), the zeta potential did decrease somewhat for the Ac_60_/siRNA complex (33 mV), indicating a modest change in the surface properties of this polyplex due to amine acetylation. This trend is consistent with the dye exclusion results (Figure 2).

**Table 2:** Particle diameter and zeta potential of dendrimer/siRNA complexes

**Sample**	**Particle diameter (nm)**	**Zeta Potential (mV)**
G5-siRNA	200.2 ± 28.1	40.2 ± 1.1
Ac_20_-siRNA	226.2 ± 7.3	38.9 ± 0.9
Ac_40_-siRNA	229.3 ± 8.4	39.7 ± 0.7
Ac_60_-siRNA	173.7 ± 8.0	33.1 ± 0.8

### Heparin induced polyplex dissociation

**Figure 3:**
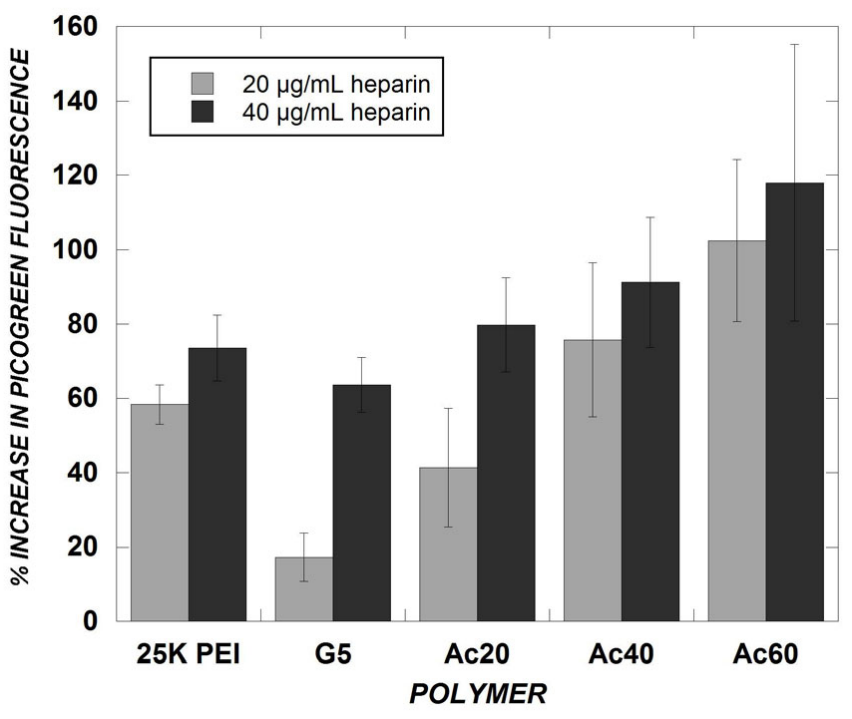
**Effect of amine acetylation on polyplex dissociation by heparin competition**. Complexes (N/P = 10) were prepared in PBS at a final siRNA concentration of 2 μg/mL, and were exposed to heparin for 60 min at 37°C. siRNA release from complexes was determined by PicoGreen fluorescence as in Figure 2. Data represent mean ± SEM (*n *= 5).

Previous work has shown that more effective cellular delivery of nucleic acids is achieved when polymers are able to release or unpackage their cargo nucleic acid easily following cellular entry [26]. To evaluate the effect of dendrimer amine acetylation on polyplex dissociation, dendrimer/siRNA complexes were challenged by exposure to heparin sulfate, an anionic competitive binding agent. Polyplexes were formed in solution at N/P = 10 and exposed subsequently to either 20 μg/mL or 40 μg/mL of heparin sulfate. After 60 minutes of incubation, PicoGreen was used to measure the amount of siRNA released from dendrimer polyplexes. As expected, more siRNA was released from complexes with increasing heparin concentration (Figure 3). Upon addition of heparin, more siRNA was released from 25 kDa polyethylenimine (25K PEI), which was included as a well-studied reference, than from G5 dendrimer, indicating that 25K PEI allows greater polyplex dissociation than G5 dendrimer. Consistent with the decrease in cationic charge density conferred by primary amine acetylation, the amount of siRNA released increased with extent of primary amine acetylation. At high fractions (40 and 60%) of amine acetylation, the polyplex dissociation of acetylated dendrimers surpassed that of 25K PEI.

### Cytotoxicity of acetylated dendrimers

**Figure 4:**
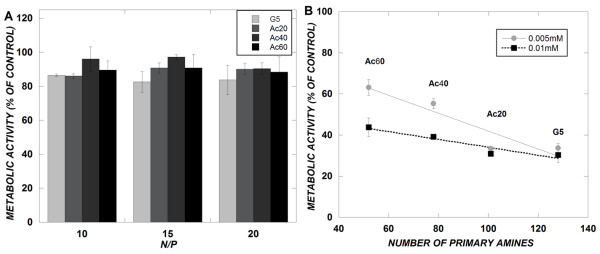
**(A and B) – Cytotoxicity of dendrimer/siRNA complexes (A) and of acetylated dendrimers (B)**. U-87 cells were exposed to dendrimer/siRNA complexes (siRNA concentration = 100 nM) or to dendrimers (0.005 or 0.01 mM) for 4 hours in OptiMEM medium prior to MTS viability assay. MTS absorbance measurements were normalized to cells receiving a mock treatment of OptiMEM medium only (100%). The unmodified, generation 5 dendrimer has 128 primary amines. Data represent mean ± SEM (*n *= 2 experiments, each measured in triplicate).

Primary amine acetylation of various cationic polymers has been shown to reduce their cytotoxicity [16,23]. The cytotoxicity of acetylated dendrimers on a malignant glioma cell line, U87, was evaluated by treatment with either dendrimer/siRNA complexes (siRNA concentration = 100 nM) or with dendrimers in OptiMEM medium for 4 hours (0.005 mM or 0.01 mM). Subsequently, cells were analyzed for cell viability using the MTS assay. Cells exposed to OptiMEM medium alone were used as positive controls. Minimal cytotoxicity was observed from treatment with dendrimer/siRNA complexes at typical cell transfection conditions (Figure 4A). However, it is also important to evaluate the cytotoxicity of the native polymers without the presence of siRNA. In this case, a dose-dependent cytotoxicity was observed by increasing the dendrimer concentrations to higher concentrations than were used for cell transfections (Figure 4B). Additionally, a linear decrease in toxicity was observed upon reducing the number of primary amines by acetylation. This trend is consistent with previous work that showed a linear decrease in cytotoxicity of a different cell type upon dendrimer amine acetylation [16].

### siRNA induced silencing of GFP in U87-d1EGFP cells

**Figure 5:**
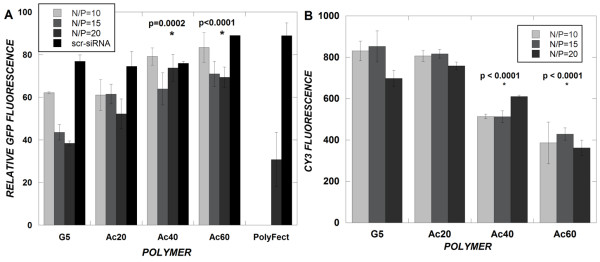
**(A and B) – SiRNA-induced GFP silencing (A) and intracellular Cy3-siRNA levels (B)**. U-87-d1EGFP (A) or non-transformed U-87 cells (B) were treated with PAMAM/siRNA complexes at a final siRNA concentration of 100 nM for 4 hours under serum-free conditions. Cells were analyzed using flow cytometry for GFP fluorescence (A) or Cy3 fluorescence (B) 24 hours after the initial treatment. PolyFect (A) was used at 5:1 wt. ratio of PolyFect:siRNA. Data represent mean ± SEM (*n *= 3) (A) or (*n *= 1 experiment, measurements performed in triplicate) (B).

We tested the effectiveness of these polymers to deliver anti-d1EGFP siRNAs to U87 cells stably expressing the d1EGFP transgene. Cells were treated with dendrimer/siRNA complexes for 4 hours at several charge ratios within the range where all dendrimers (up to Ac_60_) are able to complex the siRNA. After 24 and 48 hours, the d1EGFP fluorescence of cells was analyzed using flow cytometry. The fluorescence of polyplex treated cells was normalized to time-matched U87-d1EGFP cells that received a mock treatment of serum-free medium (positive control). SiRNAs delivered by unmodified G5 dendrimer produced significant gene silencing, manifest in reductions (up to 60% at a charge ratio of 20) of d1EGFP fluorescence after 24 hours (Figure 5a). Upon primary amine acetylation of dendrimers, a significant decrease in GFP silencing efficiency was observed, particularly for the dendrimer with the highest fractions of amine acetylation, Ac_40 _(p = 0.0002) and Ac_60 _(p < 0.0001) compared to unmodified dendrimer. The Ac_20 _dendrimer, with a modest fraction of amine modification, produced an insignificant change in GFP silencing ability as compared to the unmodified dendrimer (p = 0.28). For the G5 and Ac_20 _materials, an improved GFP silencing ability was observed upon increasing the N/P ratio of each dendrimer from 10 to 20. The extent and trends of GFP silencing using these polymers were maintained for 48 hours (data not shown). A low level of non-specific GFP silencing (~20%) was observed by all dendrimer/siRNA complexes delivering a scrambled siRNA sequence.

Having studied the ability of acetylated dendrimers to deliver siRNA to U87-d1EGFP cells to elicit a gene silencing response, we used fluorescently labeled (Cy3) siRNA to determine intracellular levels of siRNA delivered by the dendrimers to U87 cells that do not express d1EGFP. Previous work has shown that efficient gene silencing correlates to high intracellular nucleic acid levels [27]. A significant decrease in the intracellular fluorescence intensity of siRNA was observed with increasing fractions of amine acetylation (p < 0.0001 comparing Ac_40 _to G5 and p < 0.0001 comparing Ac_60 _to G5) (Figure 5B). This trend correlated closely to the decreased GFP silencing efficiency upon amine acetylation (Figure 5A).

### Confocal imaging

**Figure 6:**
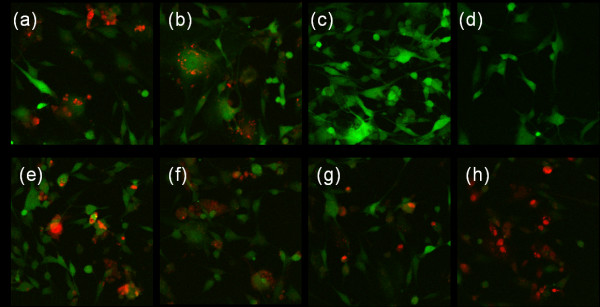
**Confocal microscopy images of Cy-5 labeled oligonucleotides delivered to U-87-d1EGFP cells**. U-87-d1EGFP cells were treated with polymer/Cy5-ODN complexes (N/P = 15). Images show U-87-d1EGFP cells in green, and Cy5-ODN in red. Images represent cells transfected with: (a) G5 PAMAM; (b) Ac_60 _PAMAM; (c) 25K PEI; (d) OptiMEM medium only; (e) G5 PAMAM + 100 μM chloroquine; (f) Ac_60 _PAMAM + 100 μM chloroquine; (g) 25K PEI + 100 μM chloroquine; and (h) Lipofectamine2000.

Confocal imaging was performed to compare the cellular distribution of nucleic acids delivered by acetylated dendrimers compared to unmodified dendrimers. As endosomal escape is a well-known barrier to efficient nucleic acid delivery [4,5], some transfections were performed in the presence of chloroquine diphosphate, a buffering agent, to identify if pH buffering is a significant barrier to efficient siRNA delivery by acetylated dendrimers. Confocal imaging indicated that oligodeoxynucleotides (ODNs) delivered by PAMAM dendrimers were sequestered into vesicles. Red ODN fluorescence appeared as isolated, punctate specks when delivered by G5 or Ac_60 _dendrimers (Figure 6a and 6b, respectively), with somewhat greater cytoplasmic distribution observed for G5 delivery. These distributions contrast with that of Lipofectamine2000, a commercially available reagent, which delivered ODN evenly throughout the cell (Figure 6h). Further, the presence of chloroquine diphosphate during transfection mediated a homogeneous distribution of ODN in a fraction of the cells when delivered by cationic dendrimers G5 (Figure 6e), Ac_60 _(Figure 6f), or 25K PEI (Figure 6g).

### Titration of acetylated dendrimers

**Figure 7:**
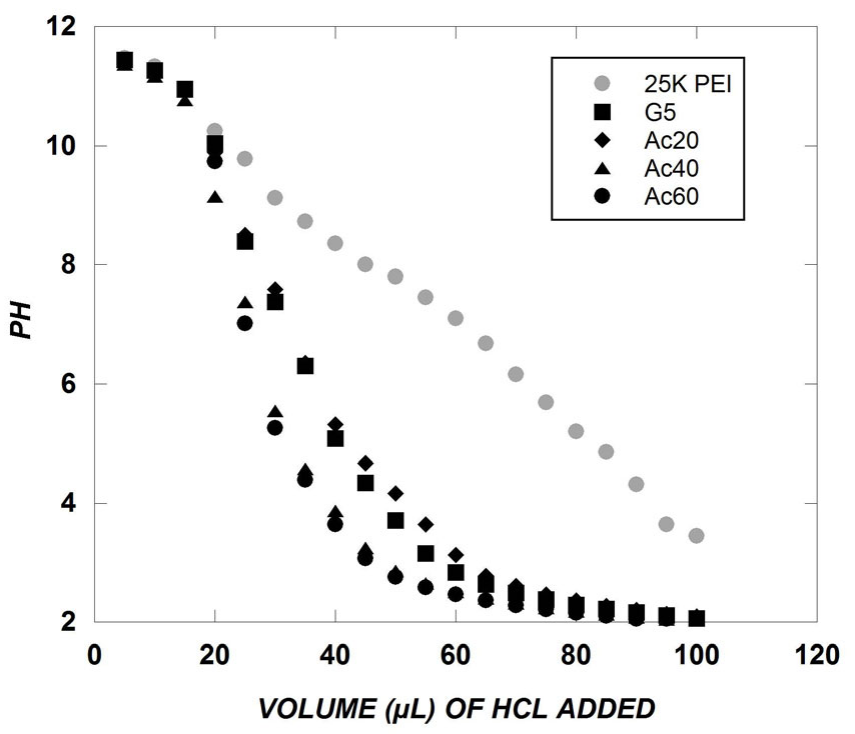
**pH titrations**. Polymers were freshly dissolved in 5 mL of PicoPure water prior to titration. Polymer solutions were first titrated to pH~11.5 using 1 M NaOH. Five microliter aliquots of 1 M HCl were added to polymer solutions and the pH was recorded.

As the cellular distribution of ODN delivered by dendrimers was improved by the presence of chloroquine in the transfection medium, it is likely that endosomal escape is a significant barrier to efficient siRNA delivery for acetylated dendrimers. Hence, pH titrations were performed to compare the buffering capacity of acetylated dendrimers. Increasing the fraction of amine acetylation caused titration curves to shift, indicating a reduction in buffering capacity (Figure 7) upon addition of acid. This trend is consistent with previous work performed with acetylation of PEI [23]. Interestingly, the titration curve showed that unmodified G5 PAMAM has a much lower buffering capacity than 25 kDa PEI. While PAMAM and PEI have similar numbers of amines per unit mass, approximately half of the PAMAM amines are secondary amines in the form of amide bonds that are not titratable.

## Discussion

One motivation behind amine acetylation of PAMAM dendrimers was to promote polymer-siRNA unpackaging in cells. That is, self-assembled vectors for siRNA delivery must associate strongly enough to remain intact during cellular binding and entry, yet they must dissociate at some point within cells to release their cargo. Previous studies have identified vector unpackaging to be an important barrier to polymeric gene and siRNA delivery [23,26,28]. Specifically, correlations have been observed between the release of DNA in competitive displacement assays in vitro and transfection efficiency in cells [26]. Furthermore, others have found that gene transfection was improved by increasing fractions of amine acetylation of PEI, and this result was attributed to decreased polymer-DNA interactions resulting in enhanced intracellular polyplex dissociation [23]. Previous work by our group has also reported a positive correlation between intracellular oligonucleotide levels (and gene silencing) and enhanced complex dissociation among various molecular weights of PEI [27].

To reduce PAMAM/siRNA interactions in this work, we employed partial amine acetylation of PAMAM dendrimers. The heparin competition assay showed that unmodified generation 5 PAMAM dendrimer (molecular weight ~28 kDa) exhibited less dissociation of siRNA than did 25 kDa PEI. Given that 25K PEI possesses a high charge density and exhibits strong association with oligonucleotides [27], the even greater resistance of G5 PAMAM to heparin dissociation can be interpreted as indicating a poor tendency of the dendrimer to dissociate from siRNA. As hypothesized, greater dissociation was observed upon primary amine acetylation of PAMAM, which surpassed that of 25 kDa PEI at a high fraction (60%) of primary amine acetylation. However, polyplex size analysis demonstrated that stable particles of high positive zeta potential were formed between acetylated dendrimers and siRNA, with a slight reduction in both at relatively high fraction (60%) of primary amine acetylation. This suggests that amine acetylation did not destabilize dendrimer/siRNA complexes in isolation, but rather facilitated the ease of dissociation of siRNA from the polymer complex in the face of an anionic competitor.

In addition to promoting polyplex dissociation by amine acetylation, we also expected to reduce cytotoxicity by partial charge neutralization. Cytotoxicity often correlates with a high charge density and is a marked problem with highly cationic PAMAM dendrimers. Previous work has shown a reduction in cytotoxicity upon amine acetylation of cationic polymers [16]. Indeed, we observed a linear decrease in cytotoxicity upon amine acetylation of PAMAM dendrimers. Thus, "tuning" of cationic polymer charge is a useful design principle in modulating both vector unpackaging and cytotoxicity.

Another important barrier to the cellular delivery of nucleic acids is their sequestration in acidic endosomes. Cationic polymers with titratable amines are thought to mediate endosomal escape by buffering in the pH range of 5–7, leading to osmotic pressure buildup and membrane permeability via the "proton sponge" effect [29]. In the current study, we found that primary amine acetylation somewhat decreased the pH buffering capacity of dendrimers by removing primary amine groups. This decrease in endosomal buffering capacity induced by amine acetylation is likely responsible for the decreased siRNA delivery, as the presence of a buffering agent appeared to improve cellular distribution of oligonucleotides delivered by acetylated dendrimers.

It is important to note that acetylation of PEI has been found to increase its ability deliver plasmid DNA [22,23] in contrast to the present study in which amine acetylation of PAMAM either did not affect (at low extents) or decreased (at high extents) the delivery of siRNA. Since siRNAs are much shorter and thus less polyvalent than plasmid DNA, it is likely that siRNA will more easily dissociate from a cationic polymer than would a large, anionic plasmid DNA, making polyplex dissociation a potentially less critical step in the delivery process [30]. Further, it has been shown that the properties of polyplexes formed by dendrimers and RNA depend significantly on the size of the RNA molecule with more stable particles being formed with large RNA molecules due to cooperative multivalent interactions[31]. These differences between polymers and their interactions with different types of nucleic acids highlight the importance of developing a more comprehensive and quantitative understanding of intracellular delivery mechanisms and their dependence on physicochemical attributes of the polymer and nucleic acid, so that data from various delivery systems and studies can be interpreted and used to rationally design nucleic acid carriers.

## Conclusion

In summary, cationic dendrimers are promising architectures for use as nucleic acid delivery vectors. Their highly organized, cationic structure with functionalizable amine groups provides them with molecular properties that are favorable for efficient nucleic acid delivery. Neutralizing a fraction of primary amines by acetylation in this study did, as expected, promote polyplex unpackaging *in vitro*. Additionally, a reduction in cytotoxicity was noticed upon acetylation. However, a reduction in pH buffering was also observed, possibly resulting in decreased siRNA delivery to tumor cells. The addition of a buffering agent chloroquine promoted favorable cellular distribution of nucleic acids delivered by dendrimers, indicating that endosomal escape is a substantial barrier to siRNA delivery by these polymers. Others efforts to modify PAMAM dendrimers for gene or siRNA delivery have also reported success in decreasing cytotoxicity but difficulties in achieving active nucleic acid delivery[15]. This observation demonstrates both the importance of endosomal buffering to siRNA delivery as well as the advantages of charge reduction including reduced cytotoxicity and enhanced vector dissociation. The next generation of dendrimers for siRNA delivery will need to integrate charge reduction of dendrimers without compromising their endosomal buffering capacity.

## Methods

### Materials

A 22 nt anti-GFP siRNA sequence identified previously [32] as an effective inhibitor of pd1EGFP expression (sense strand: 5'-UUG UGG CCG UUU ACG UCG CCG U-3', antisense strand: 3'-UGA ACA CCG GCA AAU GCA GCG G-5') was utilized in this study. A scrambled siRNA sequence (targeted against firefly luciferase) was used as a negative control (sense strand: 5'-CUU ACG CUG AGU ACU UCG A dTdT-3', antisense strand: 5'-UCG AAG UAC UCA GCG UAA G dTdT-3'). The fluorescently labeled (5' Cy3 end modified on the sense strand) and unlabeled sequences were purchased from Integrated DNA Technologies (Coralville, IA, USA). The control siRNA sequence was purchased from Dharmacon (Lafayette, CO). The lyophilized powder was resuspended to a concentration of 20 μM (unlabeled siRNA) or 50 μM (Cy3 modified sequence) according to the manufacturer's protocol before use. Generation 5 PAMAM dendrimer was purchased as a 5 wt% solution in methanol from Dendritech (Midland, MI). Branched PEI of average molecular weight 25 kDa (Item 408727), acetic anhydride, triethylamine, heparin sodium salt, and deuterium oxide (D_2_O) were purchased from Sigma. PicoGreen fluorescent dye was obtained from Molecular Probes (Eugene, OR, USA). MTS reagent was purchased from Promega (Madison, WI, USA). Unless otherwise stated, all cell culture products were obtained from Invitrogen (Carlsbad, CA, USA).

### Acetylation of PAMAM dendrimers

The molar ratio between acetic anhydride and PAMAM dendrimer was adjusted to achieve 20, 40, 60, and 80% of primary amines capped by an acetyl group. A 1:1 stoichiometric ratio of primary amines: acetic anhydride was used to achieve the desired extent of acetylation. Partially acetylated dendrimers were prepared using the following procedure: To 15 mL of anhydrous methanol in a magnetically stirred round bottom flask, 5 mL of 5 wt% PAMAM (0.214 g) in methanol was added. Triethylamine (10% molar excess to acetic anhydride) was added to the flask and was stirred for 30 minutes. The appropriate amount of acetic anhydride was then added dropwise to the reaction mixture and the reaction was carried out overnight at room temperature under an argon atmosphere. The methanol was then removed by vacuum, and the polymer residue re-dissolved in distilled water. The polymer was then dialyzed against 1 L of phosphate buffered saline (PBS) for 8 hours followed by water overnight in a 10,000 kDa cutoff Slide-A-Lyzer Dialysis cassette (Pierce Biotechnology, Rockford, IL). The samples were then lyophilized and stored at -20°C. Proton nuclear magnetic resonance (^1^H NMR) spectra were taken in D_2_O using a Varian 400 MHz or 500 MHz spectrophotometer, using the solvent as reference signal. The extent of primary amine acetylation was determined using a previously described method[24].

### PicoGreen assay for siRNA/PAMAM complexation

The complexes were prepared at various charge ratios by mixing equal volumes of PAMAM with siRNA in PBS. Charge ratios (N/P) were calculated as a ratio of the number of primary amines in the polymer, determined from ^1^H NMR spectra, to the number of anionic phosphate groups in the siRNA. The samples were then vortexed and incubated at room temperature for 15 minutes to ensure complex formation. The complexes were prepared at a final siRNA concentration of 0.2 μg of siRNA/100 μL of solution. One hundred microliters of each complex were transferred to a 96-well (black-walled, clear-bottom, non-adsorbing) plate (Corning, NY, USA). A total of 100 μL of diluted PicoGreen dye (1:200 dilution in Tris-EDTA (TE) buffer) was added to each sample. Fluorescence measurements were made after a 30 minute incubation at room temperature using a DTX800 Multimode Detector (Beckman Coulter, CA, USA), at excitation and emission wavelengths of 485 and 535 nm, respectively. All measurements were corrected for background fluorescence from a solution containing only buffer and PicoGreen dye.

### Dynamic light scattering and zeta potential

Dynamic light scattering (DLS) and Zeta Potential analyses were performed using a Malvern Instruments Zetasizer Nano ZS-90 instrument (Southboro, MA) with reproducibility being verified by collection and comparison of sequential measurements. Polymer/siRNA complexes (siRNA concentration = 100 nM, N/P = 15) were prepared using purified water (resistivity = 18.5 MΩ-cm). DLS measurements were performed at a 90° scattering angle at 37°C. Z-average sizes of three sequential measurements were collected and analyzed. Zeta potential measurements were collected at 25°C, and the Z-average potentials following three sequential measurements were collected and analyzed.

### Heparin competition assay

Complexes were prepared at a charge ratio of 10 as described above (final siRNA concentration of 0.2 μg in a total of 50 μL of solution) and were transferred to a 96-well (black-walled, clear-bottom, non-adsorbing) plate. A total of 100 μL of diluted PicoGreen dye was added to each well, followed by the addition of 50 μL of heparin solution prepared in TE buffer (pH = 8). The plate was incubated for 60 minutes at 37°C, and fluorescence measurements were then taken using the Multimode Detector plate reader. The percentage of siRNA released was calculated as described previously [27].

### Cell culture

U-87 MG cells (ATCC HTB-14) were maintained in D-MEM medium supplemented with 10% fetal bovine serum (FBS), L-glutamine, sodium pyruvate, non-essential amino acids, and penicillin-streptomycin solution. A U-87 MG cell line containing a stably integrated destabilized EGFP (d1EGFP) transgene was produced by transfecting U-87 cells with the 4.9-kb pd1EGFP-N1 plasmid (BD Biosciences Clontech, Palo Alto, CA) and maintained under constant selective pressure by G418 (500 μg/mL). All cell lines were cultivated in a humidified atmosphere of 5% CO_2 _at 37°C.

### MTS cytotoxicity assay

U-87 cells were plated at a density of 10^3 ^cells/well in a 96-well plate approximately 18 hours before the assay. Cells were exposed to 100 μL of dendrimers or dendrimer/siRNA complexes at various concentrations in OptiMEM reduced serum medium (Invitrogen, Carlsbad, CA) for 4 hours. Twenty microliters of MTS reagent were added to each sample using a multi-channel pipette, and the plate was returned to the cell incubator for 2 hours. Absorbance measurements were subsequently recorded at 490 nm using a Bio-Rad Model 680 microplate reader (Hercules, CA, USA). The values for treated samples were normalized to those for cells receiving a mock treatment of OptiMEM medium only (positive control).

### siRNA delivery assay

U87 or U87-d1EGFP cells were plated at a density of 1.5 × 10^5 ^cells/well in 12 well plates ~18 hours prior to transfection. Prior to treatment of cells, PAMAM/siRNA complexes were prepared as described above in 200 μL of PBS. PolyFect (Qiagen), a commercially available dendrimeric transfection reagent, was used as a positive control. Transfections were also performed with a scrambled siRNA sequence not targeted against GFP to account for any non-specific GFP silencing effects. Eight hundred microliters of OptiMEM medium was mixed with each sample to obtain a final siRNA concentration of 100 nM. The serum-containing culture medium was aspirated from the cells, and each well was treated with 1 mL of the PAMAM/siRNA complexes in OptiMEM medium. After a 4 hour incubation period, the transfection mixture was replaced with serum-containing culture medium and maintained under normal growth conditions until the cells were assayed for fluorescence by flow cytometry either 24 or 48 hours after initial treatment. For cells being analyzed for GFP fluorescence, unlabeled siRNA was utilized. To determine intracellular siRNA levels, non-transformed U87 cells were treated with Cy3-labeled siRNA.

### Flow cytometry

Cells were washed with PBS, detached with trypsin-EDTA, and collected in growth medium before they were pelleted by centrifugation for 3.5 min at 200 *g*, and resuspended in 150 μL PBS. Samples were maintained on ice before being subjected to flow cytometry analysis. Ten thousand cells were analyzed on a FACSCalibur two-laser, four-color flow cytometer (BD Biosciences) for GFP fluorescence (FL-1) or Cy3 fluorescence (FL-2). CellQuest software was used to acquire and analyze the results. Viable cells were gated according to their typical forward/side scatter characteristics.

### Confocal microscopy

For confocal imaging, U87-d1EGFP cells were plated into 8-well Lab-Tek Chamber Slides (Lab-Tek, Naperville, IL) at a density of 25,000 cells/well approximately 18 hours prior to transfection. For imaging purposes, complexes of PAMAM were prepared with a Cy5-labeled antisense oligonucleotide targeted against GFP[32] (ODN concentration = 100 nM) to prevent fluorescence interference between U-87-d1EGFP cells and Cy3-labeled siRNA, both of which fluoresce in the green portion of the visible spectrum. Transfections were performed in the same manner as for flow cytometry analysis. In some samples, 100 μM of chloroquine diphosphate, a buffering agent, was added to the transfection mixture. Imaging was performed at 63× magnification with a Leica LCSSB2 confocal microscope 24 hours after transfection.

### pH titrations

Either PAMAM or PEI (~4.5 mg) was freshly dissolved into 5 mL of purified water in a 10 mL beaker and was magnetically stirred. pH measurements were taken using a NMR tube micro pH probe (IQ Scientific, Carlsbad, CA). Using 1 N NaOH, solutions were adjusted to a pH of approximately 11.5. The polymer was then titrated by adding 5 μL aliquots of 1N HCl until a total of 100 μL of HCl had been added to the solution, at which point the pH had reached a constant value of pH ~2. Titrations were each performed in duplicate.

### Statistics

All statistical comparisons among treatment groups were performed using a one way ANOVA test with Tukey's all-pairs post hoc comparison test.

## Authors' contributions

CW carried out cell experiments, molecular biology assays, and drafted the manuscript. SS performed amine acetylation reactions, ^1^H NMR spectroscopy on acetylated dendrimers, and took DLS and zeta potential measurements. KU and CR conceived of the study, and participated in its design and coordination and helped to draft the manuscript. All authors read and approved the final manuscript.
